# Dr. William Seymour Morris

**Published:** 1915-11

**Authors:** 


**Dr. William Seymour Morris,** N. Y. University 1896, died at his home in Utica, Aug. 25, aged 43.

